# Salivary and serum inflammatory mediators among pre-conception women with periodontal disease

**DOI:** 10.1186/s12903-016-0306-9

**Published:** 2016-12-15

**Authors:** Hong Jiang, Yiming Zhang, Xu Xiong, Emily W. Harville, Karmin O, Xu Qian

**Affiliations:** 1 School of Public Health, Fudan University, Mailbox 175, No. 138 Yixueyuan Road, Shanghai, 200032 China; 2 Clinical laboratory, Maternal and Child Health Care Hospital, Changzhou Municipality, 16 Boai Road, Changzhou, Jiangsu Province 200032 China; 3 Key Laboratory of Public Health Safety (Ministry of Education), Fudan University, Mailbox 175, No. 138 Yixueyuan Road, Shanghai, 200032 China; 4 Department of Epidemiology, School of Public Health and Tropical Medicine, Tulane University, 1440 Canal Street, New Orleans, LA 70112 USA; 5 Department of Physiology and Pathophysiology, Faculty of Medicine, Matinoba, Canada; 6 Department of Animal Science, Faculty of Agricultural & Food Sciences, University of Manitoba, Manitoba, Canada; 7 St. Boniface Hospital Research Centre, Room 2022, 351 Tache Avenue, Winnipeg, MB R2H 2A6 Canada; 8 Global Health Institute, Fudan University, Mailbox 175, No. 138 Yixueyuan Road, Shanghai, 200032 China

**Keywords:** Periodontal disease, Inflammatory mediators, Saliva, Serum, Pre-conception women

## Abstract

**Background:**

There have been inconsistent conclusions regarding the levels of inflammatory mediators in saliva and serum among people with or without periodontal disease. Although pre-conception has been put forward as the optimal time for the periodontal treatment in order to improving pregnancy outcomes, few studies have been conducted to examine inflammatory mediators in saliva and serum among pre-conception women.

**Methods:**

Pre-conception women were recruited between January 2012 and December 2014. Women were provided with an oral health examination to detect periodontal disease. Salivary and serum samples were collected at the same of examination. Inflammatory mediators includinginterleukin-1 beta (IL-1β), IL-6, tumor necrosis factor alpha (TNF-α) and beta-glucuronidase (β–glucuronidase) were tested and analyzed among women with overall periodontal disease (*n* = 442) or moderate/severe periodontal disease (*n* = 247). Results were compared to that in women with a healthy periodontium (*n* = 91).

**Results:**

Significantly increased concentrations of inflammatory mediators of IL-1β, IL-6, TNF-α and β-glucuronidase in saliva and IL-1β, β-glucuronidase and TNF-α in serum were found among pre-conception women with moderate/severe periodontal disease, compared with women without periodontal disease. Significantly increased levels were also found in all the above saliva inflammatory mediators and in serum IL-1β and TNF-α among women with overall periodontal disease. The levels of all inflammatory mediators in saliva and almost all inflammatory mediators except IL-6 in serum significantly increased with severity of periodontal disease.

**Conclusion:**

Periodontal disease is highly associated with the elevated levels of inflammatory mediators in saliva and some mediators in serum among pre-conception women.

## Background

Periodontal disease, as a persistent infection of Gram-negative bacteria, has been found to be associated with systemic diseases such as cardiovascular diseases, stroke, diabetes mellitus and adverse pregnancy outcomes [1–3]. The periodontal pathogens and their virulence products not only damage the periodontal tissues, activating a local inflammatory response, but also trigger the systematic inflammatory response and ectopic infections through hematogenous dissemination [4]. This downstream effect by bacterial substances and host-derived inflammatory mediators is capable of initiating and promoting systemic diseases including adverse pregnancy outcomes [4, 5]. Levels of inflammatory mediators such as interleukin-1β (IL-1β), IL-6 and tumor necrosis factor alpha (TNF-α) in gingival crevicular fluid, saliva or serum are usually regarded as a reflection of host’s local and systemic inflammatory response [6–9].

Systematic reviews have shown an association between periodontal disease and increased risk of several adverse pregnancy outcomes including low birth weight, pre-term birth, gestational diabetes mellitus and preeclampsia [10, 11]. Considering the possible side effects that result from periodontal treatment during pregnancy, pre-conception has been put forward as the optimal time for the periodontal treatment in the purpose of improving pregnancy outcomes [5, 12].

Studies have been performed to explore the association between periodontal disease and systemic diseases, by detecting whether periodontal disease is linked with the levels of inflammatory mediators in mouth and blood. However, there have been inconsistent conclusions regarding the levels of inflammatory mediators among people with or without periodontal disease. Some studies showed significantly higher levels of IL-1β, IL-6 in the gingival crevicular fluid, saliva or in serum [6, 7], while others did not show such differences [8, 9]. Furthermore, very few studies examined the levels of local and systemic inflammatory mediators at the same time, leading to the weak evidence explaining the association between periodontal disease and pregnant outcomes.

This paper aims to compare the levels of local and systemic inflammatory mediators in pre-conception women with or without periodontal disease, using the baseline data of a randomized controlled trial of pre-conception treatment for periodontal disease to improve pregnancy and birth outcomes (Trial registration number: ChiCTR-TRC-12001913) [12]. The findings will contribute to understanding the associations of chronic infectious diseases such as periodontal disease and the development of systemic disease including adverse pregnancy outcomes.

## Methods

### Participants and recruitment

From January 2012 to December 2014, preconception women were recruited at the Maternal and Child Health Care Hospital, in Changzhou, China, a tertiary MCH hospital, with both pre-conception health clinic and dental clinic. The annual number of delivery is around 9000, covering more than 70% of deliveries in the city [12].

Women were approached by the research nurse in the pre-conception clinic and were invited to participate in the study if they were at least 18 years old, with the desire of being pregnant within 1 year, planning to deliver at the recruiting hospital, having more than 20 teeth, without systematic disease such as cardiovascular disease, diabetes and immunodeficiency disease, without contraindication to probing in a dental examination, and not having received periodontal treatment within the past 6 months.

After the informed consent, eligible women were interviewed to complete a questionnaire containing demographic and periodontal disease relevant information. Then women were offered with a free full-mouth dental examination for determining periodontal disease. The research received ethical approval from the Institutional Review Board of the School of Public Health, Fudan University, Shanghai, China. Written informed consent was obtained from each participant before the questionnaire survey and dental examination.

### Definition and measurement of periodontal disease

A full mouth dental examination was performed on each woman to determine periodontal disease. For each tooth, six sites (mesio-buccal, mesio-lingual, disto-buccal, disto-lingual, mid-buccal and mid-lingual) were probed by using a manual UNC-15 probe [11]. The dentist who performed dental examination had been trained and calibrated by a dentist who specializes in periodontal disease. All examination was completed by the same dentist in the hospital to avoid inter-observer measurement bias.

We used indicators combing probing depth (PD) and clinical attachment loss (CAL) over a certain threshold as a diagnosis of periodontal disease [1, 12]. Referring to the diagnostic criteria used by Offenbacher et al. [13], periodontal disease in this study was defined as: the overall periodontal disease as a presence of any site exhibiting PD > 3 mm or CAL > 3 mm; the moderate periodontal disease as the presence of 4 or more sites with PD > 3 mm; severe periodontal disease as 4 or more sites with PD ≥ 5 mm. Those who met the diagnosis criteria for periodontal disease, but not the moderate or severe degree, were categorized as the mild periodontal disease. Bleeding on probing (BOP) was defined as gingival bleeding upon probing.

### Biologic sample collection

#### Saliva collection

Saliva was collected before the dental examination. The whole expectorated saliva, without stimulation, was collected from each participant using an adapted version of the method described by Miller et al. [14]. Women were asked to firstly rinse their mouths with tap water, wait for 5 min, and then expectorate whole saliva into a clean container for 5 ml within 10 min. The research nurse moved the saliva to a sterile tube. The samples was immediately centrifuged, and the upper clear portion was kept and frozen at −80 °C.

#### Blood sample collection

At the same appointment as the dental examination, 5 ml extra blood was drawn by venipuncture. The blood samples were centrifuged for 10 min at 3000 revolutions/min and the serum put in a sterile tube and kept at −80 °C.

#### Lab test

One technologist who was blinded to the diagnosis of periodontal disease completed all the lab tests in the participating hospital. The concentration of each inflammatory mediators includingIL-1β, IL-6, TNF-α and beta-glucuronidase (β–glucuronidase) were tested using enzyme immunosorbent assays (ELISA) kits (Shanghai Biosh Biological Technology Co., Ltd, China) according to manufacturer’s directions.

### Data analysis

Women’s body mass index (BMI) was calculated as weight (kg)/height^2^ (metres^2^). Overweight was defined as BMI ≥ 25 [15]. Perceived Stress Scale with 14 items was used to measure women’s stress [16, 17]. Each item was scored 0–4 according to women’s answer and the total score ranged from 0 to 56. Pre-conception women were divided to the “high” or the “low” stress groups, according to the median score of 24.

All data were input using Epidata 3.1 and double checked for correctness. Statistical analyses were carried out using the Statistical Package for Social Sciences (SPSS) for windows version 17.0. The Pearson Chi-square test was used for categorical outcomes and *t*-test was used for continuous variables. Inflammatory mediators were checked for normality and log-transformed if significantly non-normal. Multivariate linear regression was performed for determining the association between periodontal disease and inflammatory mediators, with adjustment for women’s age, educational level, employment status, monthly family income, parity, family periodontal disease history, oral health insurance, BMI, stress score, oral hygiene. These factors were showed to be related to periodontal disease in literatures and levels of inflammatory mediators in our study [18–20]. In addition, severity of periodontal disease among pre-conception women were considered as an ordinal variable (coded as 1 through 3 representing healthy periodontium, mild periodontal disease and moderate/severe periodontal disease respectively) in the multiple linear regression model to test the linear trend.

## Result

Biological specimens, including saliva and blood samples, were taken from 533 preconception women. The average age of pre-conception women was 26 years old. Most of women (86.7%, 462/533) had college or above education level and 82.7% (441/533) were employed. Around 97.0% (517/533) women reported monthly family income was above 4000 RMB (the minimum monthly income criterion, ~USD $615). Most of women (87.1%, 464/533) were primiparas. 48.2% (257/533) women reported at least one of their family members with periodontal disease. Only 5.8% (31/533) of women reported the oral health care that was entirely or partly covered by health insurance (Table 1). A total of 80.3% (428/533) women reported tooth brushing at least twice per day. Only 8.4% (45/533) women were overweight.

**Table 1:** Characteristics of study participants

Characteristic	Preconception women (*n* = 533)			*P* ^*a^	*P* ^*b^
	Women without periodontal disease *n* (%) (*N* = 91)	Women with periodontal disease *n* (%) (*N* = 442)	Women with moderate/severe periodontal disease *n* (%) (*N* = 247)		
Age (mean ± SD)	26.53 ± 2.96	26.86 ± 3.63	26.61 ± 3.45	0.41	0.84
Education level					
Junior middle school and below	5 (5.5)	26 (5.9)	14 (5.7)	0.38^*^	0.31^*^
Senior middle school	10 (11.0)	30 (6.8)	15 (6.1)		
College and above	76 (83.5)	386 (87.3)	218 (88.3)		
Family income per month					
< 4,000RMB^c^	2 (2.2)	14 (3.2)	9 (3.6)	0.62^*^	0.51^*^
≥ 4,000RMB^c^	89 (97.8)	428 (96.8)	238 (96.4)		
Employment status					
Unemployed	16 (17.6)	76 (17.2)	43 (17.4)	0.93^*^	0.97^*^
Employed	75 (82.4)	366 (82.8)	204 (82.6)		
BMI kg/m^2^ (mean ± SD)	20.91 ± 2.48	20.80 ± 2.58	20.80 ± 2.65	0.71^*^	0.73^*^
Periodontitis family history					
No	47 (51.6)	210 (47.5)	117 (47.4)	0.74^*^	0.46^*^
Yes	11 (12.1)	53 (12.0)	23 (9.3)		
unknown	33 (36.3)	179 (40.5)	107 (43.3)		
Parity					
0	80 (87.9)	384 (86.9)	213 (86.2)	0.79^*^	0.69^*^
1 or 2	11 (12.1)	58 (13.1)	34 (13.8)		
Oral health insurance					
No	86 (94.5)	416 (94.1)	234 (94.7)	0.89^*^	0.93^*^
Yes	5 (5.5)	26 (5.9)	13 (5.3)		
Oral hygiene					
Teeth brush ≥ 2 times per day	17 (18.7)	88 (19.9)	54 (21.9)	0.79^*^	0.52^*^
Teeth brush < 2 times per day	74 (81.3)	354 (80.1)	193 (78.1)		
Smoking					
No	90 (98.9)	437 (98.9)	244 (98.8)	0.98^**^	0.93^**^
Yes	1 (1.1)	5 (1.1)	3 (1.2)		
Stress (mean ± SD)	23.80 ± 5.81	23.86 ± 5.94	24.37 ± 5.39	0.93^*^	0.40^*^

^*^Chi-square or *t*-test; ^**^ Fisher’s exact test

^a^comparison between women with overall periodontal disease and non-periodontal disease

^b^comparison between women with moderate/severe periodontal disease and non-periodontal disease

^c^1 RMB ~0.15 USD

Seventy-five of the 533 women did not have any site with BOP. The sites with BOP ranged from 1 to 108 among the rest of women, equal to 0.5–66.7% of the sites. Using the combined diagnostic criteria of PD and CAL, 82.9% (442/533) of the women were diagnosed with periodontal disease and 17.1% (91/533) as having a healthy periodontium. Among women with periodontal disease, 55.0% (243/442) was diagnosed with moderate and 0.9% (4/442) with severe periodontal disease; the remaining 44.1% were (195/442) mild (Table 2). There was no significant difference on any of socioeconomic status or periodontal relevant factors in women either with overall periodontal disease or with moderate/severe periodontal disease compared to women without periodontal disease (Table 1).

**Table 2:** Categories of periodontal disease

Indicator	*N* (%)
Women with periodontal disease	442 (82.9)
Women with moderate periodontal disease	243 (55.0)
Women with severe periodontal disease	4 (0.9%)
Women with at least one site of BOP	458 (85.9)

Table 3 shows the difference of inflammatory mediators in saliva and serum among women with periodontal disease, women with moderate/severe periodontal disease and women without periodontal disease. Compared with women without periodontal disease, all of the inflammatory mediators in saliva were significantly higher among women with overall periodontal disease or with moderate/severe periodontal disease. Serum IL-1β and TNF-α levels were significantly higher in women with overall periodontal disease compared with women without periodontal disease. Serum IL-1β, β–glucuronidase and TNF-αwere significantly higher in women with moderate/severe periodontal disease, compared with women without periodontal disease.

**Table 3:** Inflammatory mediator level among women with overall, moderate/severe and without periodontal disease

Inflammatory mediator	Women overall periodontal disease (mean ± SD) *N* = 442	Women with moderate/severe periodontal disease *N* = 247	Women without periodontal disease *N* = 91	*P* ^*a^	*P* ^*b^
Saliva					
IL-1β (pg/ml)	92.76 ± 1.16	94.63 ± 1.16	75.19 ± 1.27	<0.001	<0.001
β-glucuronidase (U/L)	699.24 ± 1.22	720.54 ± 1.21	639.06 ± 1.19	<0.001	<0.001
TNF-α (pg/ml)	44.26 ± 1.28	45.15 ± 1.02	32.46 ± 1.63	<0.001	<0.001
IL-6 (pg/ml)	52.46 ± 1.26	54.05 ± 1.25	43.38 ± 1.26	<0.001	<0.001
Serum					
IL-1β (pg/ml)	21.76 ± 2.51	21.54 ± 2.66	14.59 ± 3.13	0.002	0.004
β-glucuronidase (U/L)	361.41 ± 1.67	368.71 ± 1.70	320.54 ± 1.63	0.053	0.037
TNF-α (pg/ml)	23.34 ± 2.56	24.53 ± 2.51	15.18 ± 3.94	0.005	0.003
IL-6 (pg/ml)	18.92 ± 1.97	18.73 ± 2.01	16.12 ± 2.18	0.052	0.092

^*^ *t*-test

^a^comparison between women with overall periodontal disease and non-periodontal disease

^b^comparison between women with moderate/severe periodontal disease and non-periodontal disease

Table 4 presents ratios of the mean levels of inflammatory mediators in women with periodontal disease, women with moderate/severe periodontal disease over the levels of women without periodontal disease (reference group), after adjusting for confounding factors using multivariable linear regression. In general, the adjusted ratios showed the similar trend as compared with the crude ratios. Compared with women without periodontal disease, levels of all inflammatory mediators in saliva were 1.10–1.36 times higher; and levels in serum were 1.12–1.49 times higher in women with overall periodontal disease. The adjusted ratios were even higher when comparing women with moderate/severe periodontal disease to women without periodontal disease. Levels of all inflammatory mediators in saliva were 1.13–1.40 times higher, and levels in serum were 1.15–1.62 times higher in women with moderate/severe periodontal disease compared with the reference group.

**Table 4:** Crude and Adjusted Ratio of mean levels of inflammatory mediators in women with overall periodontal disease and women with moderate or severe periodontal disease compared with women without periodontal disease

Inflammatory mediator	Women with overall periodontal disease versus without periodontal disease (mean ± SD) *N* = 533			Women with moderate/severe periodontal disease versus without periodontal disease (mean ± SD) *N* = 338		
	Crude ratio	Adjusted ratio^a^	*P*	Crude ratio	Adjusted ratio^a^	*P*
Saliva						
IL-1β (pg/ml)	1.23	1.23	<0.001	1.26	1.25	<0.001
β-glucuronidase (U/L)	1.09	1.10	<0.001	1.13	1.13	<0.001
TNF-α (pg/ml)	1.36	1.36	<0.001	1.39	1.40	<0.001
IL-6 (pg/ml)	1.21	1.21	<0.001	1.25	1.25	<0.001
Serum						
IL-1β (pg/ml)	1.49	1.49	<0.001	1.48	1.50	0.001
β-glucuronidase (U/L)	1.13	1.12	0.048	1.15	1.15	0.025
TNF-α (pg/ml)	1.54	1.53	<0.001	1.62	1.62	<0.001
IL-6 (pg/ml)	1.17	1.18	0.039	1.16	1.17	0.077

*P* values are for adjusted ratios only

^a^Multivariable linear regression adjusted for women’s age, smoking, educational level, employment status, monthly family income, parity, family periodontal disease history, oral health insurance, BMI, stress score, oral hygiene

A significantly positive linear trend was found between the degree of periodontal morbidity and inflammatory mediators (Table 5). The levels of all inflammatory mediators in saliva and levels of all inflammatory mediators except IL-6 in serum significantly increased with severity of periodontal disease.

**Table 5:** Linear trend of severity of periodontal disease (none, mild, moderate/severe) with levels of inflammatory mediators in multivariable linear regression^a^

Saliva	Slope (95% CI)	*P*	Serum	Slope (95% CI)	*P*
IL-1β (pg/ml)	0.099 (0.079, 0.118)	<0.001	IL-1β (pg/ml)	0.160 (0.050, 0.269)	0.004
β-glucuronidase (U/L)	0.059 (0.036, 0.081)	<0.001	β-glucuronidase (U/L)	0.068 (0.011, 0.126)	0.020
TNF-α (pg/ml)	0.152 (0.117, 0.186)	<0.001	TNF-α (pg/ml)	0.216 (0.099, 0.332)	<0.001
IL-6 (pg/ml)	0.097 (0.071, 0.123)	<0.001	IL-6 (pg/ml)	0.063 (−0.016, 0.141)	0.068

^a^Multivariable linear regression adjusted for women’s age, educational level, employment status, monthly family income, parity, family periodontal disease history, oral health insurance, BMI, stress score, Oral hygiene. All inflammatory mediators were log-transformed

## Discussion

The results of our study showed, compared with women without periodontal disease, pre-conception women with moderate/severe periodontal disease had significantly increased concentrations of inflammatory mediators of IL-1β, IL-6, β-glucuronidase and TNF-α in saliva and IL-1β, β-glucuronidase and TNF-α in serum. Significantly increased levels were also found in all the above salivary inflammatory mediators and in serum IL-1β and TNF-α among women with overall periodontal disease, compared with women without periodontal disease. The positive linear trend was found between the degree of periodontal morbidity and the levels of inflammatory mediators except serum IL-6.

Socioeconomic and medical/dental factors were comparable between women with and without periodontal disease, and also between women those with moderate or severe periodontal disease and without periodontal disease. The findings showed the levels of inflammatory mediators in saliva were highly correlated with periodontal disease, and also significantly associated with the severity of periodontal morbidity. This suggests the critical role of inflammatory host response of periodontal disease, which was also indicated by previous study [21]. The IL-1β, IL-6 and TNF-α have been defined as the subpopulation of T helper 1 lymphocytes and have proinflammatory actions [22]. β-glucuronidase is an acid hydrolase found in PMN lysosomes, a marker for primary granule release produced by inflammatory cells. The increased salivary β-glucuronidase level was regarded as an important marker of periodontal destruction [23]. This might be the reason why the above inflammatory mediators were higher in women with periodontal disease compared to women with a healthy periodontium. The elevated concentrations of serum IL-1β, β-glucuronidase, TNF-α accompanying with the saliva mediators suggested periodontal disease might be one of the factors triggering a systemic inflammatory response. Studies have shown high IL-1β and TNF-α concentration in serum or amniotic fluid are associated with adverse pregnancy outcomes, including first-trimester losses, preeclampsia and gestational diabetes mellitus, and preterm birth [24–27]. However, prospective study is needed to examine whether women with periodontal disease and high inflammatory concentrations are at higher risk to develop adverse pregnancy outcomes. Our results also might indicate that an existing inflammatory response would render a woman more vulnerable to periodontal disease, similar to the interplay between diabetes and periodontal disease [28].

Our findings were consistent with the studies by Miller et al. and Ebersole et al. who also reported a significantly higher mean level of saliva IL-1β, IL-6 in the saliva and IL-1β and TNF-α in serum of people with periodontal disease [7, 29, 30], but our findings differ from the research conducted by Teles et al. and de Queiroz et al. [31, 32], who did not find a statistically significant differences of inflammatory mediators in saliva or in serum between people with periodontal disease and without periodontal disease. However, most of these studies had a relatively small sample size, usually less than 50 in the group with periodontal disease, which might led to the inadequate power to detect the difference. In addition, as Teleset al. has pointed out, the different methods of saliva collection, storage and lab testing in each study could possibly result in the different findings [31].

Our study included a relatively larger sample size than previously published studies in which patients with periodontal disease numbered less than 100 [7, 29, 32]. We analyzed the both saliva and serum samples to examine the association between inflammation response and periodontal disease. We chose saliva samples to reflect periodontal health instead of gingival crevicular fluid due to the convenient and simple collection process for saliva. In addition, the purpose of our study was to explore the degrees of host-derived inflammatory response. Saliva can reflect the overall periodontal health of the patient, rather a single point in the mouth [33, 34].

Weaknesses of the study included the high percentage of women of good social economic status and high education level among study population, which might result in the selection bias and limit the generalizability of the findings. Due to the limited funding, biological samples were notable to be analyzed in duplicate. The mediators chosen in this study were relevant both to the adverse pregnancy outcomes and periodontal disease [9, 24–27], but the involvement of mediators more specific to periodontal disease, such as matrix metalloproteinase-8 in both saliva and serum, should be considered in the future studies. The use of oral contraceptive before the recruitment was not recorded. Future studies should measure an oral health index to more thoroughly reflect oral health status.

## Conclusion

Our study demonstrated that periodontal disease is highly associated with the elevated levels of inflammatory mediators in saliva and some mediators in serum among pre-conception women. Further studies involving more specific mediators of periodontal disease are needed for explaining the association between periodontal disease and systemic inflammatory response before and during pregnancy.

## Abbreviations

BMI: Body mass index
CAL: Clinical attachment loss
IL-1β: Interleukin-1β
PD: Probing depth
TNF-α: Tumor necrosis factor alpha
β–glucuronidase: Beta-glucuronidase
